# Effect of an Enteroprotective Complementary Feed on Faecal Markers of Inflammation and Intestinal Microbiota Composition in Weaning Puppies

**DOI:** 10.3390/vetsci10070434

**Published:** 2023-07-03

**Authors:** Giorgia Meineri, Luca Cocolin, Giada Morelli, Carlo Schievano, David Atuahene, Ilario Ferrocino

**Affiliations:** 1 Department of Veterinary Sciences, School of Agriculture and Veterinary Medicine, University of Turin, Largo Braccini 2, 10095 Grugliasco, Italy; giorgia.meineri@unito.it (G.M.); david.atuahene@unito.it (D.A.); 2 Department of Agricultural, Forest and Food Sciences, University of Turin, Largo Braccini 2, 10095 Grugliasco, Italy; lucasimone.cocolin@unito.it (L.C.); ilario.ferrocino@unito.it (I.F.); 3 CeDIS (Science Information and Documentation Center), Innovet Italia Srl, Via Leonardo Da Vinci 3, 35030 Saccolongo, Italy; 4 Innovative Statistical Research Srl, Prato della Valle 24, 35123 Padova, Italy; cs@i-stat.it

**Keywords:** *Bacillus subtilis*, Calprotectin, colostrum, dog, intestinal microbiota, PEA-um, weaning, zonulin

## Abstract

**Simple Summary:**

Numerous changes occur in the gut of puppies during the delicate phase of weaning, in which they have an increased susceptibility to gastrointestinal disorders and infections. This study investigated the effects of a four-week administration of a supplement composed of ultramicronised Palmitoylethanolamide, bovine colostrum and *Bacillus subtilis* (Normalia^®^ Extra, Innovet Italia Srl, Saccolongo, Italy) on markers of gut health and microbiome of four-week-old Golden Retriever weaning puppies. No differences emerged between the control (*n* = 13) and the treated puppies (*n* = 16) with regard to growth rate and faecal quality at any study time. Faecal markers of gut inflammation were significantly decreased in treated puppies, especially at the end of the study. Few significant modifications occurred in the gut microbiome of treated puppies. We suggest that dietary integration of the study’s complementary feed can promote the intestinal health of puppies and therefore facilitate weaning.

**Abstract:**

Weaning entails numerous modifications of the intestinal structure and microbiota composition, making puppies at high risk of sickness during this delicate life stage. The aim of this study was to investigate the effects of a four-week administration of a supplement composed of ultramicronised Palmitoylethanolamide, bovine colostrum and *Bacillus subtilis* (Normalia^®^ Extra, Innovet Italia Srl, Saccolongo, Italy) on markers of gut health and microbiome of weaning puppies. Twenty-nine four-week-old Golden Retriever puppies were randomly assigned to control (CG, *n* = 13) and treated (TG, *n* = 16) groups. During the whole experimental time, there were no differences between the groups with regard to average daily gain and faecal score. In TG, faecal calprotectin and zonulin values were statistically significantly decreased compared to CG, especially at week 8 (zonulin: 42.8 ± 1.54 ng/mL and 55.3 ± 42.8 ng/mL, and calprotectin: 2.91 ± 0.38 µg/g and 5.71 ± 0.43 µg/g, in TG and CG, respectively; *p* < 0.0001 for both comparisons). Bacteria belonging to phylum *Campylobacterota* decreased (*p* = 0.04), while those referring to genera *Coprococcus* and *Pseudomonas* increased (*p* = 0.01 and *p* = 0.04, respectively). The supplementation of the tested complementary feed can promote the intestinal health of puppies and therefore facilitate weaning by lowering gut inflammation.

## 1. Introduction

Weaning is a critical stage for pets, and weaning puppies are notably at high risk of illness because of the stressors associated with weaning itself [1]. In fact, the transition from milk to solid food involves changes and modifications of the intestinal structure and the microbiome, with alterations of the digestive balance [2]. Also, changes in diet and separation from the mother occur at a time when the puppy’s immune system is not fully competent and the passive immunity provided by the bitch decreases rapidly [1,3]. Such factors increase the puppy’s susceptibility to gastrointestinal disorders and infections [2,4].

Appropriate development of neonate gut microbiota is fundamental for resisting to pathogens and decreasing the risk of mortality [5]. Puppies are exposed to a myriad of bacterial, viral and parasitic agents in the weaning phase [2]; these microorganisms frequently affect the gastrointestinal tract causing dysbiotic and diarrheal phenomena, hindering weight gain and increasing the risk of mortality within litters [6]. At the same time, the growing period is a critical window for microbiota colonisation, and potential disruptors may induce shifts in the microbiota composition that can lead to health disorders later in life [5]. Namely, enteric infections but also antimicrobial treatments may induce significant drops in the richness and diversity of the gut microbiota, as already demonstrated in adult dogs [5]. There are still few studies describing the postnatal development and modulation of the gastrointestinal microbiota related to the growth and weaning phases of puppies. However, due to growing evidence that gut microbiota plays a crucial role in the gastrointestinal health already in the early stages of life, the research for factors positively influencing it is a promising topic of research to decrease morbidity in the canine species [5].

Diarrhoea symptoms are common for dogs less than six months of age, and about 25% of puppies produce abnormal faeces during the weaning period [6,7,8]. Diarrhoea in dogs is often defined as multifactorial, because it involves nutritional factors (e.g., diet change, food type and quality), environmental stressors and infectious diseases, but also factors intrinsic to the dog like breed size and age. The digestive physiology of dogs slightly differs based on breed size: in large breed dogs, faecal moisture content is higher, soft stools are more frequent and the frequency of defecations is higher than in small breed dogs [9,10,11]. This difference may be due to higher intestinal permeability and a longer transit time, or both [12,13,14]. The same variation has been described in puppies, with large-breed puppies producing faeces of lower consistency compared to small-breed puppies [2]. Age was also shown to affect the concentrations of gut-relates markers. Indeed, lower faecal immunoglobulin A (IgA) concentrations were described in puppies below six months of age compared to adult dogs [7,15], and higher concentrations of calprotectin were found in younger puppies [8].

The research for biomarkers is a promising field for a non-invasive deeper understanding of the severity degree and/or the early diagnosis of inflammatory enteropathies [16]. Among such markers, faecal calprotectin and zonulin have gained considerable attention in canine studies in recent years. Calprotectin is released from activated neutrophils and/or macrophages that accumulate at sites of inflammation [16]. Recent studies suggest faecal calprotectin concentration may be a clinically useful marker of gastrointestinal inflammation in dogs; also, it might be useful to predict the response to immunomodulatory treatment [16,17,18]. Indeed, higher faecal calprotectin concentrations were linked to young age in puppies [8]. Zonulin is one of the few known regulators of intestinal permeability by modulating intercellular tight junctions that surround the apical portion of the enterocytes [19,20]. Zonulin has been implicated in many intestinal pathologies where mucosal integrity is impaired; in fact, an increase in the permeability of the intestinal barrier generates an early disassembly of the enterocytes and a release of zonulin, which passes directly into the intestinal lumen [21]. Among the intestinal luminal stimuli that can trigger zonulin release, exposure to bacteria and certain dietary factors were identified as powerful triggers [19]. Few studies assayed faecal markers to detect alterations in the gut functionality of puppies [7,8].

Therefore, the aim of this study was to evaluate the effects of the supplementation of a commercially available supplement intended for the improvement of the gastrointestinal health, composed of the intestinal anti-inflammatory agent palmitoylethanolamide [22] in the ultramicronised form (PEA-um), bovine colostrum and *Bacillus subtilis* (Normalia^®^ Extra), on faecal quality and faecal markers of gut inflammation (i.e., calprotectin), and intestinal permeability (i.e., zonulin) in weaning Golden Retriever puppies. Also, this study investigated the taxonomical composition of the faecal microbiome of the same puppies.

## 2. Materials and Methods

### 2.1. Animals and Feeding

This study was designed as a four-week, double-blind, randomised controlled trial. A total of 29, four-week-old (29.4 ± 0.8 d) Golden Retriever puppies from four litters of the same breeding kennel (GoldenMania, Vigone, Italy) were included. The litters were born in four different months, so each one was followed exclusively throughout the period considered (from January 2020 to July 2021). The study was approved by the University of Turin with protocol number 712/17.09.2022.

Each pregnant dam was housed in a dedicated space for the two weeks preceding the expected delivery date. The litters stayed then with their dams in heated whelping boxes (26–27 °C) of three-square meters, where they remained until the end of the trial (i.e., the eighth week of life of the puppies). Weaning occurred in 30–35 days by the third week of life. Puppies were raised in a facility meeting strict hygiene standards and complying with the requirements of the Italian legislation on animal care and protection of animals kept for farming purposes (Legislative Decree n. 146/2001) [23].

All puppies were included using the following criteria: the dogs had to be four weeks of age at the baseline; born by bitches regularly vaccinated according to the World Small Animal Veterinary Association 2016 Vaccination Guidelines [24]; dewormed with pyrantel and febantel (Drontal^®^ Puppy, Bayer AG, Leverkusen, Germany) during the second week of life; housed in the same environmental conditions during the complete assay period; and fed with the same diet throughout the study.

Also, puppies were excluded if they showed poor health conditions (e.g., prostration, dehydration, anorexia), were affected by diseases interfering with optimal growth (e.g., congenital abnormalities, megaoesophagus, pyloric stenosis, metabolic and hormonal abnormalities), were given birth by primigravida or unhealthy bitches, refused their food and were treated with any medication less than ten days prior to initiation of the study.

Before the beginning of the study, for each subject, a rapid screening test (Therapet GPC, Bioforlife, Milan, Italy) was performed and infections with *Giardia duodenalis*, Canine Parvovirus and Canine Coronavirus were excluded. Copromicroscopic examination of the faeces identified no parasites.

The puppies were randomly assigned to the control (CG) or treated groups (TG) at a 1:1 ratio using a computer-generated four-block randomization, each one corresponding to a litter. Belonging to the CG or TG was identified by customised coloured collars. Puppies in the CG received only the standardized commercial diet, while those in TG were also supplemented with a commercially available complementary feed intended for the long-term maintenance of normal intestinal function (Normalia^®^ Extra, Innovet Italia Srl, Saccolongo, Italy). For the whole study duration, each puppy in the TG was daily supplemented by the breeder with one stick of the complementary feed between meals. The stick content was mixed with 2 mL of water and immediately administered in the puppy’s mouth using an oral syringe, making sure that no product residue remained in the syringe tube. The supplement provided the following functional principles, per stick: ultramicronised palmitoylethanolamide (PEA-um), 100 mg; *Bacillus subtilis*, 1.5 billion colony-forming units; bovine colostrum, 200 mg. According to the manufacturer’s feeding instructions, animals received one stick for every 10 kg of bodyweight for 30 d at least.

All puppies were weaned using the same commercial dry food (Neobreeder-Alleva Natural puppy maxi chicken and pumpkin^®^, Diusapet SRL, Pegognaga, Italy); the kibble was balanced for growing dogs (food composition: moisture 8%, crude protein 28%, crude fat 18%, crude fibre 1.5%, ash 6.7%, Ca 1.4%, P 1.0% and metabolizable energy 4010 kcal/kg). The daily ration (g/die) was calculated based on average energy requirements (kcal/die) for newborn puppies (25 kcal × 100 g body weight), as reported in the European Pet Food Industry Federation nutritional guidelines [25], and reassessed each week after weighing the puppies. Solid food administration was divided into two meals and distributed in the morning and late afternoon.

### 2.2. In Vitro Digestibility Assay

The digestibility of the diet with and without the supplement was determined in vitro with the Daisy incubator II (Ankom, Macedon, USA). The incubator is equipped with four rotating digestion vessels that come stirred at a constant and uniform temperature inside a controlled temperature chamber. Each jar can hold up to 23 filter bags with samples, one blank without the sample and an enzymatic solution. Diets with and without the supplement were weighed (0.5 ± 0.01 g) in triplicate in filter bags Ankom F57, heat sealed, put in the jar with enzymes and buffer solution, and digested. The enzymes used consisted of pepsin (P7125, Sigma Aldrich, Darmstadt, Germany), pancreatin (P1500, Sigma Aldrich, Darmstadt, Germany) and bile salt. The phosphate buffer consisted of the acid (KH2PO4) and its conjugate base (K2HPO4). During digestion, the vessels were stirred at a constant temperature of 39 °C. At the end of incubation, the bags were washed, the disappearance of the substance was measured dry. The in vitro sample digestibility (IVD%) value was calculated as the difference between the initial sample and the undigested one; the obtained result was divided by the initial mass sample and multiplied by 100.

### 2.3. Data and Faecal Samples Collection

Each puppy was visited weekly in the morning inside its kennel by the same investigator, from the fourth to the eighth week of life. At each visit, puppies’ body mass (BM) was recorded using an electronic scale in standard conditions. The average daily gain (ADG) was calculated for each subject as follows:

ADG = (BM of week (*n*) − BM of week (*n* − 1))/7.

During the visits, the operator waited for each subject to defecate spontaneously in order to evaluate faecal consistency using a 13-point scale specific for puppies during the weaning period, as previously described [2]. Faeces with a score ≤5 were classified as abnormal [2]. Stool samples (≈10 g) were also collected by using a sterile spatula immediately after spontaneous defecation at week 4, week 6 and week 8. The samples were separated into two aliquots, one for the measurement of faecal calprotectin and zonulin and one for faecal microbiota characterisation. Fresh faeces were placed in sterile polypropylene tubes at +4 °C and transferred to −20 °C within one hour (Nutritional Chemistry Laboratory of the Department of Veterinary sciences, University of Turin, Turin, Italy) until being processed by blind investigators within two months.

### 2.4. Faecal Calprotectin and Zonulin Concentration

Faecal calprotectin concentration was determined via a species-specific enzyme-linked immunosorbent assay (ELISA) developed and analytically validated at the Gastro-intestinal Laboratory of Texas A&M University [17]. Following thawing, the faecal samples (aliquots of 1.0 ± 0.3 g) were prepared and analysed according to the test manufacturer’s protocol (Bühlmann Laboratories AG, Schönenbuch, Switzerland). The optical density was determined at 450 nm using a microplate spectrophotometer reader.

Zonulin concentration was determined by using an ELISA kit (Immundiagnostik AG). The assay used the competitive binding technique. Biotinylated zonulin tracer was added to the samples, standards and positive and negative controls as a competitor to the sample’s own zonulin. The intensity of the colour was inversely proportional to the zonulin concentration in the sample. Samples were read using a 450 nm microplate spectrophotometer reader. 

### 2.5. Faecal DNA Extraction and 16S rRNA Amplicon Sequencing Analysis

Following the manufacturer’s instructions, the total DNAs from faecal samples (200 mg) were extracted using the RNeasy Power Microbiome KIT (Qiagen, Milan, Italy), skipping steps 11–13 to obtain also DNA. After extraction, for 30 min, treatment at 37 °C with RNase (Thermo Scientific, Waltham, MA, USA, 10 mg/mL) was performed. DNA was then quantified using the QUBIT dsDNA Assay kit (Life Technologies, Milan, Italy) and standardized at 5 ng/μL. DNA was used for PCR amplification spanning the V3-V4 region of the 16S rRNA [26], using the primer 16S Amplicon PCR Forward Primer = 5′TCGTCGGCAGCGTCAGATGTGTATAAGAGACAGCCTACGGGNGGCWGCAG. 

16S Amplicon PCR Reverse Primer = 5′GTCTCGTGGGCTCGGAGATGTGTATAAGAGACAGGACTACHVGGGTATCTAATCC, as per protocol by Illumina (https://support.illumina.com/documents/documentation/chemistry_documentation/16s/16s-metagenomic-library-prep-guide5044223-b.pdf; accessed on 13 June 2023). Library preparation and sequencing by MiSeq instrument (Illumina, San Diego, CA, USA) were carried out following the 16S Metagenomic Sequencing Library Preparation instruction.

After sequencing, raw reads were analysed by QIIME 2 v. 2022.2.0 [27]. Cut adapter plugin of QIIME 2 was used for primers and adapters filtering. Denoising was performed by DADA2 algorithm v. 2022.2.0 [28], removing low-quality bases, chimeric sequences, and sequences shorter than 300 bp by using the DADA2 denoise-paired plug in of QIIME 2. Amplicon sequence variants (ASVs) were then used for taxonomic assignment using the QIIME feature-classifier plugin against the Greengenes 16S rRNA gene database v.13.5. Taxonomy assignment was double-checked on BLAST suite tools. QIIME 2 diversity script was used to perform alpha diversity analysis [27].

### 2.6. Bioinformatics and Statistical Analysis

The metadata collected during recruitment were entered in a spreadsheet (Excel, Microsoft) and subjected to descriptive analysis. Significant associations between categorical variables (i.e., sex, faecal score) were determined by Fisher’s exact test; changes in the outcome scores among time points were compared with the Kruskal–Wallis test. Differences in BM, ADG, faecal calprotectin and zonulin concentration based on sex, litter, time and treatment and their interaction were assessed using a generalized linear mixed model (GLMM; SAS version 9.4) with post-hoc Tukey’s pairwise comparisons. The puppy was also included as a random effect to account for repeated measurements on the same animal. Differences in diet digestibility were assessed using GLMM (SAS version 9.4). Differences were considered significant for a *p*-value of less than 0.05.

Spearman’s rho coefficient was calculated to evaluate the correlation between faecal score and faecal calprotectin and zonulin concentrations.

About microbiota profiling, statistical analysis of bacterial proportions and alpha diversity (explored using observed richness (sobs), Chao1, Shannon and Pielou indexes) was carried out by performing a GLMM analysis (SAS version 9.4). Due to non-normally distribution of proportion, data were previously transformed into ranks. The model included the effects of sex, litter, time and treatment, and the interaction between time and treatment; the puppy was included as a random effect; *p*-value < 0.05 was considered significant. 

## 3. Results

### 3.1. Growth of Participant Puppies

Thirteen CG puppies (males: *n* = 8, 62%; females: *n* = 5, 38%) and 16 TG puppies (males: *n* = 8, 50%; females: *n* = 8, 50%) entered the study. There was no inequality in the distribution of sexes by group (*p* = 0.71) or litter (*p* = 0.36). All puppies were healthy during the study and no side effects (e.g., vomiting, diarrhoea) were recorded in either group. No food waste was found in any of the stalls throughout the study period, and there was no change in food consumption.

**Figure 1:**
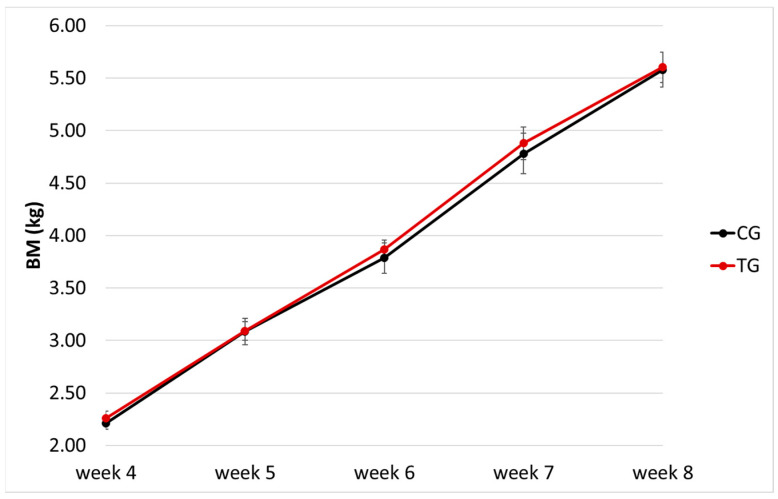
Growth chart (body mass, BM, x week of age) of the puppies enrolled (*n* = 29); different colours indicate the control (CG, *n* = 13) and treatment (TG, *n* = 16) groups.

At week 4, the average BM of the CG group was 2.21 ± 0.21 kg (range, 1.91–2.70 kg) and 2.26 ± 0.27 kg (range, 1.87–2.75 kg) for the TG; there was no significant difference in BM distribution between the two groups (*p* = 0.95). After four weeks (week 8), the average BM of the CG group was 5.58 ± 0.59 kg (range, 4.73–6.71 kg) and 5.60 ± 0.58 kg (range, 4.68–6.72 kg) for the TG; again, there was no significant difference in the distribution of BM between the two groups (*p* = 1). The growth curve of the puppies is shown in Figure 1; no differences in BM were found at any timepoint (*p* > 0.9 for all). Overall, puppies in the TG did not show major variations in BM and ADG compared to the CG (*p* = 0.49 and *p* = 0.33, respectively).

A significant effect of sex was found on BM, as female puppies were lighter than males (average difference: −0.174 ± 0.056 kg; *p* = 0.005; however, this did not have an effect on the ADG (*p* = 0.67). Also, puppies born from different dams displayed significant variations in their BM (*p* < 0.0001) and in their ADG (*p* = 0.005). Details on each puppy’s characteristics and growth can be found in Table S1.

### 3.2. Diet Digestibility

No difference was found in the digestibility of the diet alone (86.66 ± 2.14%) and combined with the supplement (85.66 ± 0.20%; *p* = 0.35).

### 3.3. Faecal Score

Faecal score of TG and CG showed no significant difference at baseline (*p* = 0.88). However, faecal score distribution at week 4 differed based on litter (*p* < 0.0001), as puppies from one particular dam produced softer faeces irrespective of the experimental group they belonged to (CG = 4; TG = 5).

**Figure 2:**
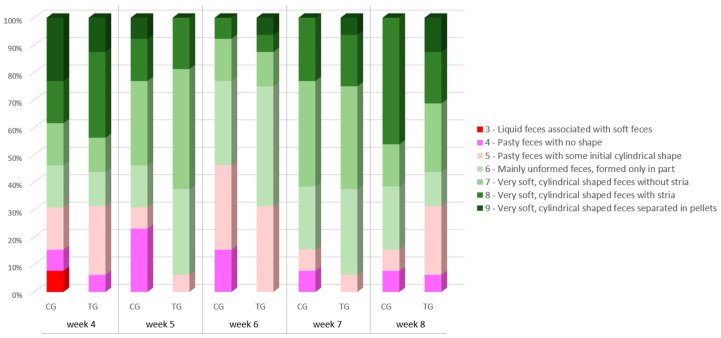
Faecal scores of the puppy dogs in control (CG, *n* = 13) and treatment group (TG, *n* = 16). Different colours indicate different scorings.

No difference in the faecal score was detected between any weekly timepoint and baseline for both experimental groups (*p* > 0.05 for all). Faecal score distribution between the two experimental groups (Figure 2) showed no difference also at any weekly timepoint (*p* > 0.05 for all). However, at weeks 5, 6 and 7 (i.e., after 1, 2 and 3 weeks of supplementation, respectively) no faecal score <5 could be detected in TG puppies, while CG groups had 23%, 15% and 8% of faeces scored 3 or 4 (i.e., liquid or pasty faeces with no shape). Each puppy’s faecal score can be found in Table S1.

### 3.4. Faecal Calprotectin and Zonulin Concentration

**Figure 3:**
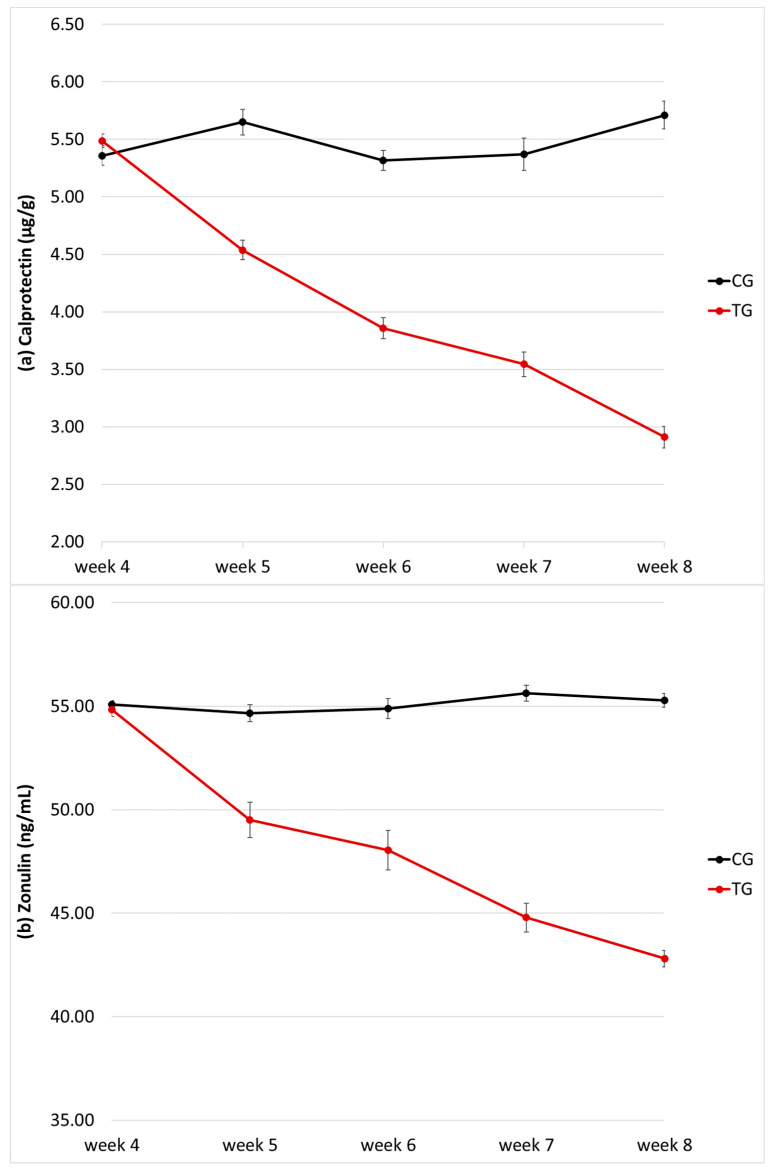
Faecal calprotectin (**a**) and zonulin (**b**) concentration of the puppy dogs in control (CG, *n* = 13) and treatment group (TG, *n* = 16). Data are given as µg/g and ng/mL, respectively.

Faecal calprotectin concentration did not differ between the two groups at baseline (5.36 ± 0.31 µg/g and 5.49 ± 0.23 µg/g in CG and TG, respectively; *p* = 0.96), but it decreased significantly with time only in the TG (*p* < 0.0001; Figure 3a); values are shown in Table 1. At week 8, faecal calprotectin concentration in the TG was almost half of that detected in the CG (2.91 ± 0.38 µg/g and 5.71 ± 0.43 µg/g, respectively) and such difference was significant (*p* < 0.0001; Figure 3a). A significant interaction (*p* < 0.0001) between time and treatment was also recorded. Faecal calprotectin concentration showed no variation based on the sex of the puppy (*p* = 0.35) or the litter it belonged to (*p* = 0.14). No correlation was found between faecal calprotectin concentration and faecal score (ρ = 0.01, *p* = 0.87).

Faecal zonulin concentration did not differ between the two groups at baseline (55.1 ± 0.62 ng/mL and 54.8 ± 1.30 ng/mL in CG and TG, respectively; *p* = 1), but it decreased significantly with time only in the TG (*p* < 0.0001; Figure 3b); values are shown in Table 2. At each time, faecal zonulin concentration in the TG was significantly lower of that detected in the CG, especially at week 8 (42.8 ± 1.54 ng/mL and 55.3 ± 42.8 ng/mL, respectively; *p* < 0.0001; Figure 3b). A significant interaction (*p* < 0.0001) between time and treatment was also recorded. Faecal zonulin concentration showed no variation based on the sex of the puppy (*p* = 0.13), but an effect was seen considering the litter it belonged to (*p* < 0.0001).

No correlation was found between faecal zonulin concentration and faecal score (ρ = −0.04, *p* = 0.64). A significant correlation was found between faecal calprotectin and zonulin concentration (ρ = 0.77, *p* < 0.0001).

Each puppy’s calprotectin and zonulin values can be found in Table S1.

### 3.5. Faecal Microbiota Composition

A total of 386.867 reads after denoising were used for downstream analysis with 4446 reads/sample on average and a sample coverage >99% (Table S2). The 16S rRNA gene sequencing results showed the microbial composition across the time and group (Table S3). Overall, the dominant phyla were *Firmicutes*, *Proteobacteria*, *Fusobacteriota* and *Bacteroidota* as shown in Table 3. Those phyla distributions, together with *Campylobacterota*, differed significantly among litters (*p* ≤ 0.01). The frequency of *Proteobacteria* and *Actinobacteriota* differed between treatment groups (*p* = 0.036) and study times (*p* = 0.038), respectively, while *Campylobacterota* frequency lowered significantly with supplementation (mean ± SD: 0.0 ± 0.04 in CG and 0.1 ± 0.19 in TG at week 4; 0.5 ± 1.28 in CG and 0.0 ± 0.00 in TG at week 8; *p* = 0.042). 

**Figure 4:**
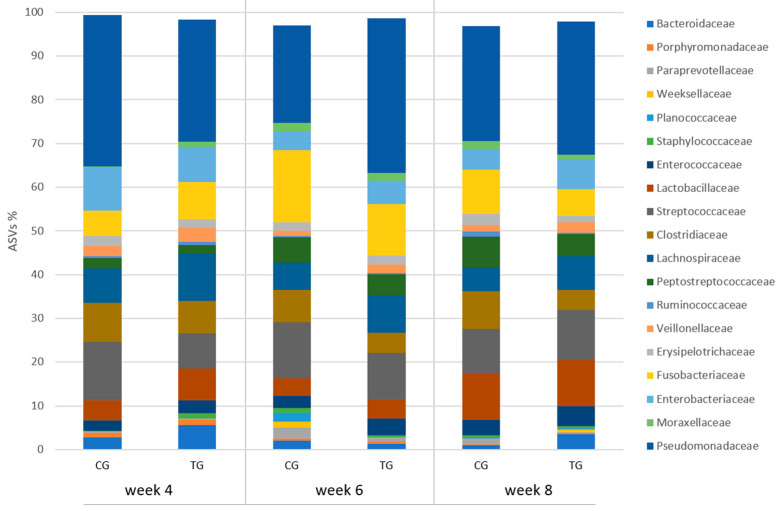
Relative frequency of ASVs at family level in faecal samples of the puppy dogs in control (CG, *n* = 13) and treatment group (TG, *n* = 16); families with relative frequency >1% are included.

Puppies fed with the tested supplement showed the presence of family *Moraxellaceae* at a relative frequency remaining constant from 0.02% at week 4 to 0.03% at week 8, while it increased in the control animals (0% vs. 0.07%; *p* = 0.007); also, *Pseudomonadaceae* increased in TG from 24.5% at week 4 to 31.23% at the end of the study (*p* = 0.039), while it almost halved in the untreated puppies (38.1% vs. 19.9%). Family distribution is illustrated in Figure 4. All genera whose distribution showed a significant interaction treatment × time are reported in Table 4.

Alpha diversity indices for all samples are summarized in Table 5. At the phylum level, alpha diversity as described by sobs and Chao 1 was significantly affected by treatment × time (*p* < 0.05); at the genus level, the same outcome was described by the Shannon index.

The litter had a significant effect on Shannon and Pielou indexes both at phylum (*p* = 0.0001 and *p* < 0.0001, respectively) and genus level (*p* = 0.016 and *p* = 0.006, respectively), and also on sobs at the genus level (*p* = 0.039). ASV-based measures of phylogenetic diversity showed no significant differences (Table S3).

## 4. Discussion

The present study showed that feeding weaning puppies a balanced diet supplemented with PEA-um, *Bacillus subtilis* and bovine colostrum (Normalia^®^ Extra, Innovet Italia Srl, Saccolongo, Italy) was associated with significantly enhanced gut health. This finding is particularly valuable as puppies are very vulnerable during this early life stage, and stress and nutritional changes are common reasons for digestive disorders, especially in breeding kennels where the risks of illness are even increased. No side effects were observed in the treated puppies, which showed regular food consumption and daily weight gain; indeed, there were no differences in body mass of treated puppies compared to the control subjects at any experimental time. This suggests that diet rationing was carried out correctly and that supplementation did not adversely affect growth-related parameters, nor the diet digestibility, as confirmed by digestion analysis.

Nonpharmacological interventions such as the administration (as treatment or prevention) of nutraceuticals have gained increasing attention over the last decade for their potential applications in companion animal gastroenterology [29]. In fact, such agents may exert biological effects similar to those by conventional treatments (i.e., anti-inflammatory, immunomodulatory and antimicrobial), but not competing for the same molecular targets and causing little or no side effects [29]. 

To study the effect of the supplementation, we measured the faecal score and two specific faecal biomarkers of inflammation and gut permeability. Alterations of faecal appearance in puppies are frequent and, in addition to representing an externalization of the change in diet and environmental stress conditions, they can also be a symptom of gastrointestinal disorders caused by digestive pathogens [2,6]; the latter was excluded at enrolment as the puppies underwent screening for main infectious diseases. A scale from 1 (liquid) to 13 (hard stool) previously validated by Grellet et al. was used for the assessment [2]. Despite the lack of statistically significant difference between the two groups at all timepoints, the treated puppies had better faecal scores overall. Despite no animal showing diarrhoea proper, it should be remarked that puppies from one particular dam produced softer faeces throughout the study so that all statistical analyses were influenced by the outliers; also, the simple size made it not possible to test the significance after outliers’ exclusion.

Remarkable differences between groups were found in faecal calprotectin and zonulin concentrations. Calprotectin contributes about 60% to the protein content of the neutrophil cytosol. Any disturbance of the mucosal architecture due to the inflammatory process causes the escape of neutrophils, and therefore of calprotectin, into the intestinal lumen and its subsequent excretion in the faeces [30]. Previous studies have reported a significant correlation between calprotectin levels and inflammatory states such as IBD [31,32] or chronic inflammatory enteropathies [17,33]. Therefore, the decrease in faecal calprotectin levels assessed in puppies treated with the study complementary feed could indicate a reduction in inflammation and a more stable intestinal environment. Faecal calprotectin levels detected in this study are in line with previous data; in the study by Grellet and colleagues [8], faecal calprotectin concentration obtained from healthy weanling puppies ranged from 2.9 to 59.5 µg/g (median: 10.5 µg/g); in the study by Heilmann et al. [33], values obtained from healthy adult dogs ranged from 3 to 159 µg/g (median: 4 µg/g). The study by Grellet and colleagues [8] also provided data collected from puppies whose faeces quality was judged abnormal; those values were higher on average (range: 2.9–421.4 µg/g, median: 15.2 µg/g) and were significantly affected by the age of the subjects, namely weanling puppies between 5 and 8 weeks of age showed higher mean values than older puppies.

Zonulin is a 47 kDa protein released by several cell lines in the body, including epithelial cells lining the small intestine. Zonulin acts on the intestinal tight junction, and several studies documented its overexpression in pathological conditions that alter intestinal permeability [19,21,34]. Though serum zonulin originates from several different tissues, faecal zonulin seems to be the most closely associated with intestinal permeability, as zonulin secreted from the intestinal barrier may leak into the lumen [20,21]. A recent study by Rossi et al. [20] found that faecal zonulin in ten dogs evaluated for gastrointestinal conditions (e.g., GI dysmotility, irritable bowel syndrome) was 141.56 ± 72.67 ng/mL (allegedly, the unit was not reported in the study at the time of writing). In our study, the treated puppies showed significantly lower faecal zonulin levels during any single experimental time (except for baseline) compared to the control animals. Similar to calprotectin, such a result may define better intestinal integrity and permeability in the treated puppies. However, these data must be interpreted carefully as there are still no studies in veterinary medicine regarding the expected levels of zonulin at weaning to the best of the authors’ knowledge. Also, compared to the abovementioned studies, calprotectin and zonulin concentrations showed smaller deviations from the average in our work; sample homogeneity (in terms of genetics, breed, life stage and environment) may have played a role in shaping such narrow ranges.

The effects of the administered supplementation can be ascribed to the combination of three ingredients, namely palmitoylethanolamide (PEA), bovine colostrum and *Bacillus subtilis*, with proven enteroprotective, immunomodulatory and eubiotic (i.e., stimulating the growth of a healthy microbiota) properties. 

PEA is a N-acylethanolamine endogenously produced from membrane glycerophospholipids [35]; it functions as a pro-homeostatic mediator against inflammation and tissue damage, by down-modulating the activity of immune cells (e.g., mast cells, macrophages) and glial cells [36,37,38]. Aside from playing a key role in maintaining intestinal homeostasis under normal conditions, enteric glial cells may respond to gut injury with an excessive release of neurotrophins, growth factors and cytokines resulting in the recruitment and further activation of immune cells, such as macrophages, neutrophils and enteric mast cells [39,40]. The anti-inflammatory effects of PEA depend on its ability to activate, directly and indirectly, receptors belonging to the extended endocannabinoid system (i.e., the endocannabinoidome) [41], like peroxisome proliferator-activated receptor-alpha (PPAR-α) [22]. PEA-mediated activation of PPARα reduces nitric oxide production, neutrophil influx and the expression of proinflammatory mediators in the colon mucosa [42,43]. Experimental studies with mice demonstrated that PEA administration is able to reduce intestinal inflammation and normalise intestinal motility [22,44], provided it is formulated in bioavailable form, e.g., ultramicronised PEA [45]. Accordingly, a study performed on human volunteers showed the ability of PEA to prevent inflammation-induced hyperpermeability of the gut [43]. Recent evidence also suggests a link between endocannabinoid-like modulators (e.g., PEA) and gut microbiota homeostasis [46].

Bovine colostrum is an immunomodulating compound rich in nutrients and bioactive peptides, including growth factors and immunoglobulins (Ig) that can inhibit the colonization of the intestine and the production of biological toxins by harmful microorganisms [39,40,47]. Substantial amounts of orally ingested bovine colostrum survive passage through the stomach to remain intact and active in the lower parts of the intestine [39]. It has been demonstrated that colostrum is helpful in preventing and limiting diarrhoea of different origins, as well as reversing infection-induced inflammation of the digestive tract, possibly through the improvement of mucosal integrity, tissue repair and direct antimicrobial action [48,49]. A randomized placebo-controlled study carried out with 70 weanling puppies demonstrated a greater improvement in faecal quality in the colostrum-supplemented group [47]. Moreover, another study showed increased faecal IgA levels (suggestive of the enhanced immune response) and improved gut microbiota diversity as well as stability in dogs receiving dietary bovine colostrum [39].

The endospore-forming *Bacillus subtilis* is one of the few probiotic bacteria admitted in animal feeds [50]. Its form’s advantage over probiotics given as vegetative cells is that spore formation provides long-term survival even in extreme environmental conditions, i.e., high temperature, acidic pH [51]. *B. subtilis* is a normal constituent of human and canine microbiota [52], and it was shown to improve faecal quality and gut health markers in dogs [53,54,55,56,57,58]. Supplementation with *B. subtilis* improved faecal consistency and faecal odour, promoted the synthesis of short-chain fatty acids, i.e., propionic acid and butyrate, and decreased faecal ammonia content in dogs [56,57,59]. Moreover, a greater bacterial diversity was found in dogs fed *B. subtilis* [57,59] and a lower CIBDAI score (Canine Inflammatory Bowel Disease Activity Index) was recorded in dogs affected by chronic inflammatory enteropathy consuming a diet fortified with *B. subtilis* spores [59].

Weaning is a crucial step in the establishment and development of puppies’ gut bacterial population, as the shift from milk to solid food fosters the abundance and activity of certain bacterial groups [5]. As long as the most determining events have occurred (i.e., oxygen homeostasis, diet transition, environmental changes), microbiota composition gets more stable following the ageing of the puppy [5]. In the current study, both groups showed an evolution of the gut microbiota over time, and the supplementation did not seem to remarkably influence the overall development of the puppies’ gut populations despite the presence of components with prebiotic and probiotic actions. However, at the phylum level a significant decrease was found in *Campylobacterota* in the treated compared to the control group. This may be considered a positive finding as the increase in *Campylobacterota* has been associated with the development of gastrointestinal diseases [60]. The phylum *Campylobacterota* is a miscellaneous group of bacteria comprising both commensal and pathogenic genera, with the last being well-known etiological agents of relevant infections and serious associated diseases in humans [61]. Within the *Campylobacterota* phylum, the genus *Helicobacter* can be divided into two groups: gastric *Helicobacter* species (which colonize the gastric mucosa of animals) and enterohepatic *Helicobacter* species (EHH, which colonize the intestinal mucosa and/or the hepatobiliary tract). During the last years, several EHH have become increasingly important because of their association with human gastrointestinal diseases [62]. According to a recent study, dogs with chronic gastrointestinal signs are frequently infected by “non-*Helicobacter pylori* helicobacters” (NHPH), and an association between infection and lymphoid follicular hyperplasia was demonstrated [63]. In animal models, a reduction in genus *Helicobacter* was achieved through probiotic treatment already in two weeks [60]. On the other hand, *Campylobacter* species also belong to the *Campylobacterota* phylum. Because of the frequent carriage of pathogenic *Campylobacter* species (e.g., *C. jejuni*) by pets, they can constitute a significant source of human exposure to *Campylobacter* [64] and may also contribute to the spread of antimicrobial resistance due to their close contact with people. Some studies showed that approximately 6% of human enteric campylobacteriosis is transmitted from pets, and direct evidence of the transmission of fluoroquinolone-resistant *C. jejuni* between humans and pets living in the same households has already been shown [65].

At the genus level, the treated puppies showed a significant increase in *Coprococcus* which is another favourable outcome: the genus *Coprococcus* belongs to a group of anaerobic cocci that are known to produce butyrate, the preferred energy source of the colon epithelial cells which contributes to the maintenance of the intestinal barrier functions and has immunomodulatory and anti-inflammatory properties [66]. A low abundance of *Coprococcus* was said to promote the development of IBD in many studies carried out with humans and animal models [67,68,69]. Butyrate-producing *Coprococcus* bacteria were also consistently associated with a higher quality of life in humans and their depletion was common in major depressive disorders [70], suggesting a major role in the gut–brain axis balance.

This is not the first time that a decreased abundance of *Faecalibacterium* was found in weaning puppies [71,72], contrary to the authors’ expectations. Also, previous studies found that *Faecalibacterium* spp. bacteria were reduced in the faeces of dogs suffering from acute and chronic diarrhoea [73,74], undergoing antibiotic therapy [75], or with chronic inflammatory enteropathy [76]. However, some authors were unable to detect significant differences between control dogs and dogs with chronic enteropathy in samples of the intestinal mucosa [77]. Being both *Faecalibacterium* and *Coprococcus* major butyrate-producer genera of the order *Clostridiales*, phylum *Firmicutes* [5], one could argue that *Faecalibacterium* may have decreased at the expense of *Coprococcus* in the treated puppies to maintain a similar functionality.

About the significant over-representation of genera *Psychrobacter* and *Pseudomonas*, we cannot exclude an effect by prolonged storage as suggested by Weese and Jalali [78] or soil contamination during sampling. In fact, *Psychrobacter* spp. is a cold water, Gram-negative, nonmotile bacterium that is a predominant commensal on aquatic animals, and whose distribution is ubiquitous in both marine and terrestrial environments [79]. To the best of the authors’ knowledge, *Psychrobacter* spp. was never supposed to belong to the core gut microbiome of the canine species; it was identified nonetheless on the skin [80,81], mouth [82] and eye of dogs [83]. The genus *Pseudomonas* is also widely recognized as being among the most diverse and ubiquitous bacterial taxa, with 242 currently validated species which inhabit diverse habitats [84]. In one study, *Psychrobacter* and *Pseudomonas* spp. were significantly more abundant on the skin of malodourous dogs [80]. Also, both cold-resistant genera have been associated with food spoilage [85]. The increased presence of *Pseudomonas* was observed in dogs with idiopathic inflammatory bowel disease [86], thus representing a potential negative finding in this study. In a recent study by Del Carro and colleagues [87], *Psychrobacter* and *Pseudomonas* spp. were isolated from a sample of dam colostrum, and *Psychrobacter* spp. was found in more than 30% of the puppies’ meconium collected at birth. 

Finally, as confirmed by both taxonomic composition and alpha diversity, we distinguished a higher similarity in the faecal microbiota of puppies within the same litter, even if the dams belonged to the same kennel; this finding is in agreement with previous studies [87,88]. Also, with regard to alpha diversity, this is expected to increase or settle in growing puppies [5,89], but decreased richness measures (i.e., sobs and Chao1) were observed in the treated puppies in the current study. However, recent evidence demonstrated that a reduction in richness values could be the consequence of dietary interventions focused on gut health including prebiotics and probiotics [90,91].

All puppies included in this study were born and grown in the same kennel. They were thus exposed to the same management, food type and distribution and environmental conditions. This made it possible to study the effect of the supplementation in different litters, minimizing the impact of the mentioned factors. However, this study has potential limitations that must be addressed. First, coprophagia and subsequent gastrointestinal upset cannot be excluded in some individuals, as this is a common issue in young dogs. Second, a larger sample size would have diminished the impact of inter-individual variability in gut microbiota composition on biostatical analyses, plausibly providing alternative outcomes regarding statistical significance. Last, although faecal samples were collected with particular care, soil contamination cannot be ruled out.

## 5. Conclusions

The results of this study suggest that feeding a supplement for long-term intestinal health composed of PEA-um, bovine colostrum and *Bacillus subtilis*, may have beneficial effects on the gut health of weaning puppies, which are experiencing a stressful event due to changes in the diet. In fact, the complementary feed was well tolerated by the animals and decreased two indicators of intestinal damage, suggesting a protective role during early growth. Some intestinal microbial populations were also positively affected by the supplementation, yet more research is needed to validate the meaning of the current results.

## Figures and Tables

**Table 1:** Faecal calprotectin concentration of the puppy dogs in control (CG, *n* = 13) and treatment group (TG, *n* = 16). Data are given as µg/g; *p*-values are determined by post-hoc Tukey–Kramer pairwise comparisons.

	CG		TG		*p*
	Mean ± SD	Range	Mean ± SD	Range	
week 4	5.4 ± 0.31	4.7–5.8	5.5 ± 0.23	5.1–5.8	0.96
week 5	5.7 ± 0.41	5–6.0	4.5 ± 0.34	4–5	<0.0001
week 6	5.3 ± 0.31	4.9–5.9	3.9 ± 0.36	3.2–4.7	<0.0001
week 7	5.4 ± 0.51	4.7–6.1	3.5 ± 0.43	2.9–4.1	<0.0001
week 8	5.7 ± 0.43	4.9–6.3	2.9 ± 0.38	2.4–3.7	<0.0001

**Table 2:** Faecal zonulin concentration of the puppy dogs in control (CG, *n* = 13) and treatment group (TG, *n* = 16). Data are given as ng/mL; *p*-values are determined by post-hoc Tukey–Kramer pairwise comparisons.

	CG		TG		*p*
	Mean ± SD	Range	Mean ± SD	Range	
week 4	55.1 ± 0.62	53.7–56.0	54.8 ± 1.30	51.1–56.1	1
week 5	54.7 ± 1.51	52.0–57.0	49.5 ± 3.39	44.2–55.5	0.0018
week 6	54.9 ± 1.75	51.3–57.2	48.1 ± 3.82	40.1–54.9	0.0002
week 7	55.6 ± 1.39	53.4–58.7	44.8 ± 2.77	40.30–49.9	<0.0001
week 8	55.3 ± 1.20	53.9–57.8	42.8 ± 1.54	40.1–45.4	<0.0001

**Table 3:** Phyla frequency of the puppy dogs’ faecal microbiota in control (CG, *n* = 13) and treatment group (TG, *n* = 16) at 4, 6 and 8 weeks of age. Data (% of sequences) are given as median and first and third quartiles (Q1–Q3). Values of 0 counted as zero, while 0.0 counted as below 0.05.

	Week 4		Week 6		Week 8	
	CG	TG	CG	TG	CG	TG
*Actinobacteriota*	0 (0–0.1)	0.0 (0–0.3)	0.2 (0.0–0.8)	0.1 (0.0–0.4)	0.5 (0.3–0.8)	0.1 (0.0–1.7)
*Bacteroidota*	3.3 (0.7–5.2)	4.7 (0.2–11.1)	2.6 (0.6–12.8)	1.1 (0.2–2.4)	1.2 (0.6–2.9)	0.4 (0.1–6.9)
*Campylobacterota*	0 (0–0)	0 (0–0.1)	0 (0–0)	0 (0–0.0)	0 (0–0)	0 (0–0.0)
*Firmicutes*	42.3 (24.1–61.3)	47.8 (34.5–57.7)	34.2 (26.4–66.0)	38.4 (29.2–59.1)	53.6 (37.5–61.7)	52.6 (43.0–59.8)
*Fusobacteriota*	6.1 (0.1–7.7)	6.0 (3.3–12.2)	10.8 (2.8–30.1)	7.0 (1.0–22.0)	8.9 (3.3–13.5)	3.6 (1.3–9.2)
*Proteobacteria*	44.3 (26.1–57.7)	37.0 (24.7–46.4)	30.4 (20.2–33.6)	40.1 (32.0–50.4)	28.4 (18.5–40.7)	38.8 (30.4–46.5)

**Table 4:** ASVs at genus level of the puppy dog faecal microbiota in control (CG, *n* = 13) and treatment group (TG, *n* = 16) at 4, 6 and 8 weeks of age, which showed a significant interaction treatment × time at GLMM analysis. Data (% of sequences) are given as median and first and third quartiles (Q1–Q3).

	Week 4		Week 6		Week 8	
	CG	TG	CG	TG	CG	TG
Unclassified *Muribaculaceae* (f) (*p* = 0.020)	0.00 (0.00–0.00)	0.00 (0.00–0.01)	0.00 (0.00–0.00)	0.00 (0.00–0.01)	0.00 (0.00–0.03)	0.00 (0.00–0.00)
*Coprococcus* (*p* = 0.011)	0.04 (0.00–0.11)	0.00 (0.00–0.00)	0.03 (0.00–0.08)	0.00 (0.00–0.03)	0.00 (0.00–0.03)	0.02 (0.00–0.07)
*Faecalibacterium* (*p* = 0.034)	0.00 (0.00–0.00)	0.00 (0.00–0.00)	0.00 (0.00–0.07)	0.00 (0.00–0.02)	0.07 (0.04–0.22)	0.00 (0.00–0.00)
*Psychrobacter* (*p* = 0.034)	0.00 (0.00–0.00)	0.00 (0.00–0.09)	0.00 (0.00–0.16)	0.00 (0.00–0.00)	0.00 (0.00–0.34)	0.00 (0.00–0.18)
*Pseudomonas* (*p* = 0.037)	38.09 (13.41–50.51)	24.51 (17.05–31.29)	21.30 (13.41–24.91)	36.33 (26.26–41.29)	19.94 (12.93–37.62)	31.15 (19.64–38.46)

**Table 5:** Alpha diversity measures at phylum and genus level of the puppy dog faecal microbiota in control (CG, *n* = 13) and treatment group (TG, *n* = 16) at 4, 6 and 8 weeks of age. Data show the interaction treatment × time at GLMM analysis and are given as mean and standard deviation (SD).

	Week 4		Week 6		Week 8	
	CG	TG	CG	TG	CG	TG
Phylum						
Observed richness (sobs) (*p* = 0.016)	4.4 ± 0.65	5.0 ± 0.82	5.2 ± 0.69	4.8 ± 0.83	5.4 ± 0.96	4.6 ± 0.50
Chao1 (*p* = 0.015)	4.4 ± 0.65	5.0 ± 0.82	5.2 ± 0.73	4.8 ± 0.83	5.4 ± 0.96	4.6 ± 0.50
Shannon index (*p* = 0.216)	0.9 ± 0.24	1.0 ± 0.20	1.0 ± 0.30	1.0 ± 0.22	1.0 ± 0.25	0.9 ± 0.17
Pielou (*p* = 0.630)	0.6 ± 0.16	0.6 ± 0.14	0.6 ± 0.19	0.6 ± 0.11	0.6 ± 0.11	0.6 ± 0.11
Genus						
Observed richness (*p* = 0.058)	22.8 ± 5.42	26.1 ± 8.81	32.3 ± 7.63	28.1 ± 5.92	33.9 ± 7.60	28.3 ± 8.14
Chao1 (*p* = 0.102)	23.5 ± 5.58	26.3 ± 8.84	33.0 ± 7.30	28.7 ± 6.01	34.3 ± 7.91	29.0 ± 8.59
Shannon index (*p* = 0.014)	1.8 ± 0.42	2.0 ± 0.47	2.2 ± 0.42	2.0 ± 0.32	2.2 ± 0.45	2.0 ± 0.34
Pielou (*p* = 0.080)	1.8 ± 0.42	2.0 ± 0.47	2.2 ± 0.42	2.0 ± 0.32	2.2 ± 0.45	2.0 ± 0.34

## Data Availability

Data are available upon request to the authors. The sequence data have been deposited in the National Centre for Biotechnology Information (NCBI) Bioproject database with accession number PRJNA961925.

## Supplementary Materials

The following supporting information can be downloaded at: https://www.mdpi.com/article/10.3390/vetsci10070434/s1, Table S1: Puppies’ characteristics, growth rate, faecal score, faecal calprotectin and faecal zonulin concentrations. Table S2: Coverage of detected ASVs. Table S3: taxonomic reconstruction and distribution of the bacterial populations encompassed by all analysed samples, based on single individual, and also on time and group, rarefaction plot and ASVs-based diversity measures.
